# Reminiscences Of The First His Bundle Electrography In India

**Published:** 2002-04-01

**Authors:** CA Nambiar

**Affiliations:** Former Professor of Cardiology, Medical College Calicut, Kerala, India

We spent two hectic years in AIIMS  in 1972 and ''73 while undergoing our training for DM Cardiology. Dr Rajan Manjuran and myself were the two candidates and we worked day and night, but thoroughly enjoyed it as it was so exciting and educative. The cutting edge remarks by the inimitable Professor Sujoy B Roy, the meticulous precision of Prof M.L.Bhatia and the tender auscultatory sessions on infants with Prof Raj Tandon all are still fresh in mind. Another fascinating aspect of those days was the frequent visits by the leading innovators in cardiology and cardiac surgery.

Dr Onkar Narula was making a huge wave those days with his work on His Bundle Electrocardiography and Corrected Sinus Node Recovery Time. He visited us in late 1972 or early 1973,probably the latter date. Till then venous catheterizations (including pacing) were done by open cut down. I remember the first time we used venous sheath through right femoral vein. Dr Bhatia was the operator and I was assisting him and Dr Savithri Srivasthava was the senior Registrar. With Dr Narula showing the way we did the first His Bundle Recording and I analysed the whole roll manually as was the method those days. We had to improvise a junction box as the recording equipment did not have a proper connecting accessory at that time. After Dr Narula left we did His Bundle recording on many more patients and undertook a study. This was published as: Effect of acute digitalization on His Bundle Intervals and Corrected Sinus Node Recovery Time in young adults. M.L.Bhatia, C.Ashokan Nambiar, S.Shrivastava and Sujoy B. Roy: Indian Heart Journal: Vol-2:1977 [1].The article was received for publication on 29/11/1973 as acknowledged underneath, but strangely it took a long time to come in print. I have preserved some of the cath rolls and reprint of the article.
